# Of the Causes, Nature and Treatment of Palsy and Apoplexy: Of the Forms, Seats, Complications and Morbid Relations of Paralytic and Apoplectic Diseases

**Published:** 1850-12

**Authors:** 


*Of the causes, nature and treatment of Palsy and Apoplexy: of the forms, seats, complications and morbid relations of paralytic and apoplectic diseases.* By James Copland, M. D. F. R. S., &c., &c. Philadelphia, Lea & Blanchard, 1850.

Dr. Copland, the author of the above work, is so favorably known to the medical profession of this country, as an industrious and able writer, that it is scarcely necessary to say a word in commendation of any work he may deem appropriate for publication. Few men have written more, and none better than he has, on various medical subjects. The publication of his Medical Dictionary, was of itself, a herculean undertaking, requiring an immense amount of industry and information, and he has performed his task with so much ability, and with so little dogmatical prejudice, that every one will freely accord to him the highest distinction, not only as a medical writer, but as a physician of vast experience and sound judgment.

"A considerable part of the above work was published many years ago in the first and third volumes of the Author's Dictionary of Practical Medicine, and several of the chapters on the connection of Paralytic and Apoplectic seizures, with other disorders, formed the Croonian Lectures for 1846 and 1847, at the Royal College of Physicians." The close connection between apoplexy and paralysis, as cause and effect, constituted a good reason for the publication of these several articles, in a connected form. The two diseases should be studied together, or the young practitioner will hardly be able to appreciate their true pathology and treatment. In the language of Dr. Copland, it is necessary to describe "not merely the primary and uncomplicated forms of disease, but also the several associated or complicated states, in which each malady most frequently comes under the observation of the physician, and he was not the less convinced of the propriety of recognising alliances and connections between diseases, too often described as distinct species, extreme features of difference being chiefly or only insisted upon and intimate relations generally disregarded."

In the arrangement of the work, Dr. Copland considers first, the simple and primary varieties of palsy; next the uncomplicated forms of apoplexy; third, the complicated states of apoplexy and paralysis; fourth, the causes and pathology of these diseases; lastly, the treatment of the different varieties &c. Each one of these points is discussed with such practical precision, that no one can peruse the work without acquiring the most accurate information in regard to the symptoms, causes, pathology and treatment of these two diseases.

Without entering into a detailed analysis of the several sections of which the above work is composed, we commend it to the medical public as one of the most valuable works on Apoplexy and Paralysis with which we are acquainted.

